# Bone mineral density in Jamaican men on androgen deprivation therapy for prostate cancer

**DOI:** 10.1186/1750-9378-6-S2-S7

**Published:** 2011-09-23

**Authors:** Belinda F Morrison, Ingrid E Burrowes, William D Aiken, Richard G Mayhew, Horace M Fletcher, Marvin E Reid

**Affiliations:** 1 Department of Surgery, University of the West Indies, Mona, Jamaica; 2 Department of Obstetrics and Gynaecology, University of the West Indies, Mona, Jamaica; 3 Sickle Cell Unit, University of the West Indies, Mona, Jamaica

## Abstract

**Background:**

Androgen deprivation therapy (ADT) has been reported to reduce the bone mineral density (BMD) in men with prostate cancer (CaP). However, Afro-Caribbeans are under-represented in most studies. The aim was to determine the effect of androgen deprivation therapy (ADT) on the bone mineral density (BMD) of men with prostate cancer in Jamaica.

**Methods:**

The study consisted of 346 Jamaican men, over 40 years of age: 133 ADT treated CaP cases (group 1), 43 hormone-naïve CaP controls (group 2) and 170 hormone naïve controls without CaP (group 3). Exclusion criteria included metastatic disease, bisphosphonate therapy or metabolic disease affecting BMD. BMD was measured with a calcaneal ultrasound and expressed in S.D. units relative to young adult men (T score), according to the World Health Organization definition. Patient weight, height and BMI were assessed.

**Results:**

Mean ± sd, age of patients in group 1 (75± 7.4 yrs) was significantly greater than groups 2 and 3 (67 ± 8.1 yrs; 65±12.0 yrs). There was no significant difference in weight and BMI between the 3 groups. . The types of ADT (% of cases, median duration in months with IQR) included LHRH (Luteinizing hormone releasing hormone) analogues (28.6%, 17.9, IQR 20.4), oestrogens (9.8%, 60.5, IQR 45.6) anti-androgens (11.3%, 3.3, IQR 15.2) and orchiectomy (15.7%, 43.4, IQR 63.9). Unadjusted t score of group 1, mean ± sd, (-1.6± 1.5) was significantly less than group 2 (-0.9±1.1) and group 3 (-0.7±1.4), p <0.001. Ninety three (69.9%), 20 (45%) and 75 (42%) of patients in groups 1, 2 and 3 respectively were classified as either osteopenic or osteoporotic (p<0.001). Adjusting for age, there was a significant difference in t scores between groups 1 and 2 as well as between groups 1 and 3 (p<0.001). Compared with oestrogen therapy and adjusting for duration of therapy, the odds of low bone mineral density (osteopenia or osteoporosis) with LHRH analogue was 4.5 (95%CI, 14.3 to 3.4); with anti-androgens was 5.9 (95%CI, 32.7 to 5); with orchiectomy was 7.3 (95%CI, 30 to 5.8) and multiple drugs was 9.2 ((95%CI, 31 to 7.1).

**Conclusions:**

ADT is associated with lower BMD in Jamaican men on hormonal therapy for prostate cancer.

## Introduction

Jamaica is a middle income country, situated in the tropics, with a population of ~2.7 million and GNI per capita of 4,870 [1]. Prostate cancer is the leading cancer in Jamaican men, with an annual age-specific incidence rate of 65.5 per 100,000 [2]. It is also the commonest cause of male cancer-related deaths in Jamaica [3]. Despite the introduction of PSA screening in Jamaica in 1991, the disease continues to be detected at an advanced stage [4-6]. The use of androgen deprivation therapy is widespread in Jamaica. Common agents used are steroidal and non-steroidal anti-androgens, leutenizing hormone releasing hormone (LHRH) analogues, conjugated oestrogens and bilateral orchiectomy.

Androgen deprivation therapy is associated with several long-term complications [7]. Reduction in bone mineral density (BMD) typically occurs within 6-12 months of use of androgen deprivation therapy [8,9]. However prolonged use of androgen deprivation therapy is associated with an increased fracture risk [10]. This correlates with overall increased morbidity and reduced survival [11]. Most studies on the effects of ADT on BMD were performed with samples that were dominated by Caucasian men from high income countries. Recent evidence suggests an ethnic variation in the effects of ADT on BMD. For example, Japanese men exposed to ADT have low rates of osteoporosis [12] In general, men of African descent have higher bone mass than Caucasian men, adjusting for age [13]. Further, Afro-Caribbean men living in tropical environments and in lower income countries are likely to have different environmental exposures compared with Caucasian men from high income countries, which can influence BMD and the change in BMD with age and therapy. We therefore sought to determine in a sample of Jamaican men the effects of ADT on BMD.

## Methods

### Sample

The sample was recruited from men attending the urology clinic at the University Hospital of the West Indies, Mona, Jamaica from October 2008 – June 2009. Men were eligible for recruitment if they were >40 years. The men were divided into 3 groups:

Group 1- men with a histological diagnosis of prostate cancer, non-metastatic, treated with androgen deprivation therapy (surgical or chemical castration).

Group 2- hormonal naiive men with prostate cancer (Treated with radical prostatectomy, external beam radiation or active surveillance).

Group 3- hormone naiive men without prostate cancer.

Exclusion criteria included clinical or radiological evidence of bone metastases, bony metabolic disease e.g. Paget disease, hyperthyroidism, Cushing disease; renal failure, prior bisphosphonate therapy or drugs affecting bone metabolism.

Consent was obtained from the Ethics Board Committee, Faculty of Medical Sciences. All patients provided written informed consent. The nature of androgen deprivation therapy was determined as well as the duration of use. Types of androgen deprivation therapy included steroidal and non-steroidal anti-androgens, LHRH analogues, conjugated oestrogens and bilateral orchiectomy. Patient co-morbidities were determined as well as a history of smoking and alcohol use. Bone mineral density was measured with a calcaneal ultrasound. Weight (kg) was measured using a beam balance, to the nearest 0.1kg and height (m) was measured using standard technique with a staidometer, to the nearest 0.1cm [14]. Weight and height were measured and Body mass index (BMI) was calculated as weight (kg)/ height (m) ^2^.

### Bone mineral density assessment

Bone mineral density was evaluated quantitatively using the Achilles Express calcaneal ultrasound machine. BMD was expressed in standard deviation units relative to young adult men (T score) according to the World Health Organization definition (Normal: t score -1.0 or greater, Osteopenia- t score between -1.0 and -2.5 and Osteoporosis- t score -2.5 or lower).

### Statistical analyses

Data are expressed as frequencies, means with sd or medians with interquartile ranges as appropriate. For categorical outcome variables, differences in proportions between groups were tested with the Chi square statistic. For normal distributed continuous outcome variables, differences between groups were tested by analysis of variance (ANOVA) and for skewed continuous outcome variables differences in distribution, were tested with the Kruskal-Wallis procedure. Post Hoc comparisons following ANOVA were performed using Scheffe’s method and post hoc comparisons following the Kruskal-Wallis procedure were done using Wilcoxon rank-sum test. Multivariate regression analyses were used to assess differences in BMD by group adjusting for age and anthropometry and logistic regressions were performed to estimate the risk of low BMD (osteoporosis or osteopenia) adjusting for age, BMI or duration of drug therapy. The data was analyzed with the Stata statistical software for Windows™ version 10(College Station, TX 77845, USA).

## Results

### Patient characteristics

Table 1 summarizes the patient characteristics. A total of 346 black Jamaican men were enrolled in the study. Group classifications were as follows: group 1 (133 patients), group 2 (43 patients) and group 3 (170 patients). Mean ± sd, age of patients in group 1 (75±7.4 yrs) was significantly greater than groups 2 and 3 (67 ± 8.1 yrs; 65±12.0 yrs). There was no significant difference in weight and BMI between the 3 groups.

Table 2 summarizes the frequency and duration of the different modalities of ADT used. 34.6% of the sample had exposure to multiple types of ADT. Of the patients with single agent exposure, LHRH analogues were the most frequently used method of ADT (28.6%); however conjugated oestrogens had the longest duration of use (60.5 mos).

**Table 1:** Patient characteristics

Variables	Group 1	Group 2	Group 3	p value
N	133	43	170	
Age (yrs)	75±7.4^a,b^	67±8.1	65±12.0	<0.0001
Weight (kg)	70.4±10.8	71.5±11.6	72.8±14.4	0.4
Height (cm)	166.5±6.8^b^	169.4±6.8	169.6±7.3	0.006
BMI (kg/m^2^)	25.4±3.9	25.0±3.9	25.2±4.2	0.8
T score	-1.6±1.5^a,b^	-0.9±1.1	-0.7±1.4	<0.0001*
Normal Bone density†	40 (30)	23 (55)	95 (58)	158 (46)
Osteopenia†	56 (41)	18 (40)	59 (33)	133 (37)
Osteoporosis†	37 (29)	2 (5)	16 (9)	56 (17)

Values are mean±s.d.

†values are counts (%).

Group 1- prostate cancer patients receiving androgen deprivation therapy.

Group 2- prostate cancer patients, hormonally naiive.

Group 3- non prostate cancer patients; hormonally naiive.

BMI- Body Mass Index.

p value comparison of means by analysis of variance (ANOVA) with post hoc comparisons by Scheffe.

a- Group 1 vs Group 2: p<0.005.

b- Group 1 vs Group 3: p<0.005.

c- Group 2 vs Group 3: p<0.005.

* p value comparison of ranks by Kruskal Wallis procedure with post hoc comparisons by Wilcoxon test.

a- Group 1 vs Group 2: p<0.005.

b- Group 1 vs Group 3: p<0.005.

c- Group 2 vs Group 3: p<0.005.

**Table 2:** Types of Androgen deprivation therapy

Drug Class	Frequency (%)	Duration in months*		T score†	Osteopenia (%)	Osteoporosis (%)
		Median	25^th^ and 75^th^ percentile			
Multiple drugs	34.6	17.4	9.3, 56.2	-1.8± 1.4	33	46
LHRH analogues	28.6	17.9	9.3, 29.6	-1.5± 1.5	42	24
Oestrogens	9.8	60.5	22.2,67.8	-0.7± 1.6	38	7
Anti-androgens	11.3	3.3	0.7, 15.9	-0.9± 2.2	33	27
Orchiectomy	15.7	43.4	15.7, 79.6	-1.8± 1.4	43	38

Values are percents.

† Values are means ± s.d.

*p <0.001: Overall comparisons of medians/ranks across drug classes.

Abbreviations- LHRH- Luteinizing hormone releasing hormone.

Multiple drugs- Exposure to 2 or more different types of androgens.

### BMD results

**Figure 1:**
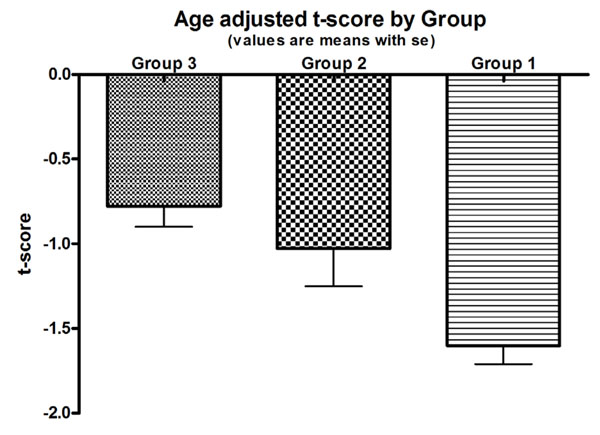
Age-adjusted t score by Group.

**Figure 2:**
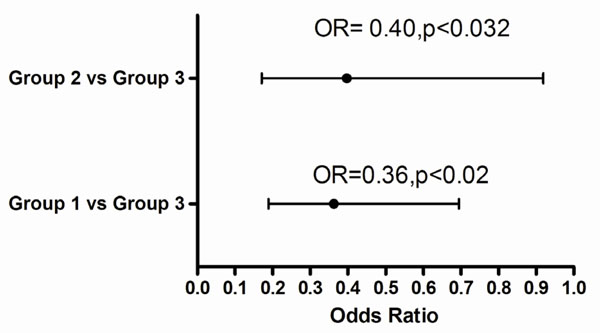
Odds Ratio for low bone mineral density, adjusting for age and BMI. Low BMD; Osteoporosis and osteopenia. Group 1- prostate cancer patients receiving androgen deprivation therapy. Group 2- prostate cancer patients, hormonally naiive. Group 3- non prostate cancer patients; hormonally naiive.

Unadjusted t score of group 1, mean ±sd, (-1.6± 1.5) was significantly less than group 2 (-0.9±1.1) and group 3 (-0.7±1.4), p <0.001. Adjusting for age, there was a significant difference in t scores between group 1 and groups 2 as well as between group 1 and 3 (p<0.001) Figure 1. Compared with group 3, and adjusting for age and BMI, the odds of low bone density (osteoporosis or osteopenia) was 0.36, (95%CI, 0.33 to 0.17) for group 1 and was 0.40 (95%CI, 0.52 to 0.22) Figure 2

**Figure 3:**
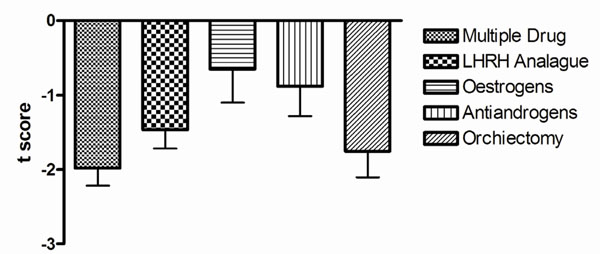
Age-adjusted t score by drug class.

**Figure 4:**
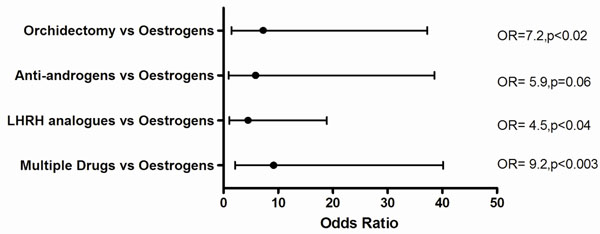
The risk of low BMD (osteoporosis or osteopenia) adjusting for duration of therapy. Reference drug therapy =oestrogens.

Similarly adjusting for age, the mean BMD scores for all drug classes were in the osteopenic range (<-1.0) except for oestrogens (mean ±se; -0.65±0.45) and anti-androgens (-0.88 ±0.40) Figure 3. Of the total sample of men, 46% had a normal BMD. Osteoporosis or osteopenia was detected in (93) 70%, (20) 45% and (75) 42% in groups 1, 2 and 3, respectively, p<0.001. Compared with oestrogen therapy and adjusting for duration of therapy, the odds of low bone mineral density (osteopenia or osteoporosis) with LHRH analogue was 4.5 (95%CI, 14.3 to 3.4); with anti-androgens was 5.9 (95%CI, 32.7 to 5); with orchiectomy was 7.3 (95%CI, 30 to 5.8) and multiple drugs was 9.2 ((95%CI, 31 to 7.1) Figure 4. There were no fractures noted during the study period.

## Discussion

In this study, we report that men with prostate cancer treated with ADT had significantly lower BMD compared with hormone naïve men with prostate cancer and controls without prostate cancer. All drug classes, except oestrogens and anti-androgens resulted in a reduced BMD. Additionally, we also found that a low BMD was common in men with prostate cancer who were hormonally naiive.

Several studies have documented progressive osteoporosis after treatment of prostate cancer with ADT [8,15,16]. The difference was apparent from as early as 6-12 months of treatment. Longer duration of ADT therapy increases the risk of bone complication [17]. In our study, the rates of osteopenia compare favourably with those seen in other studies, however the prevalence rates of osteoporosis in all three groups studied our series were lower than the prior documented studies. [17]. Morote et al detected osteopenia and osteoporosis in 45.3% and 41.5% of 53 men respectively, with prostate cancer receiving prolonged ADT compared to 43.8% and 28.1% of 57 men respectively, with prostate cancer, not treated with ADT, p (osteoporosis) =0.162 [15]. Wei et al documented osteopenia and osteoporosis in 38% and 50% of 32 men respectively, treated with ADT for over 1 year, compared to 38% and 25% of 8 men who were hormonal naiive [16]. The high prevalence of low BMD in men with prostate cancer, prior to the commencement of ADT is in keeping with a prior cross-sectional study of 41 men with prostate cancer [18].

Many studies regarding BMD have men of African ethnicity under-represented. The determinants of BMD in persons of African descent is poorly studied and understood. Results of the Tobago Bone Health Study revealed that BMD was 10-20% higher in Afro-Caribbean males than in United States non-Hispanic black and white males [13]. Postulated differences in BMD due to ethnicity may be related to lifestyle factors, genetic differences, peak bone mass acquisition and bone geometry [13]. Important lifestyle factors include increased physical activity which may confer an increase in bone density. Several studies have suggested that African ethnicity may be protective against skeletal related complication in use of ADT, in men with prostate cancer [11,17].

The clinical consequences of low BMD in men with prostate cancer are quite grave. Fracture risk is increased in men with prostate cancer, treated with ADT [10]. Skeletal complications lead to impaired quality of life, impaired mobility, increased medical costs and reduced overall survival [11,19,21].

Our study had several limitations. We were not able to analyze various metabolic, lifestyle or dietary factors that could modify BMD loss. History of alcohol intake and smoking were not reliably obtained in all patients, and could not therefore be analyzed with BMD. However, weight and BMI, which are known to affect BMD, did not affect t scores.

Our method of measurement of BMD utilized quantitative ultrasound. Calcaneal ultrasound utilizes an ultrasound probe which is placed on the heel to measure BMD. It is portable, inexpensive and does not utilize ionizing radiation. BMD is commonly measured using DEXA [22]. However, DEXA is expensive, not portable and not universally available. Several studies have suggested comparable results of DEXA and calcaneal ultrasound in other groups of patients [23,24].

As seen from prior reports, our study suggests a protective effect of oestrogens on BMD [25]. Historically, oestrogens were used as the primary modality of androgen suppression, however due to the thrombo-embolic and cardiovascular toxicity; this agent fell out of favour. In Jamaica however, oestrogens continue to be used as first and second line therapy in patients with prostate cancer, without these significant adverse effects. Due to the oncologic effectiveness of this agent and its proven bone protective effects; it may be considered the preferred treatment of choice for ADT in a resource poor country such as Jamaica. Due to the study design; we were unable to determine the effect of duration of ADT and appearance of changes in BMD. There were several patients who had exposure to different modalities of ADT during the study period. This may have been due to changes in drug availability, increased drug cost or rising PSA. However, the results were consistent, even after excluding these patients.

To the best of our knowledge, this is the first report of the effect of ADT on BMD of patients of African descent with prostate cancer. Due to the widespread use of this agent in Jamaica, and the morbidity of osteoporosis and fractures, we would recommend similar local screening guidelines in Jamaica. We would also like to perform further prospective studies to compare the efficacy of calcaneal ultrasound and DEXA as screening methods in a similar subset of patients.

## Conclusions

ADT is associated with lower BMD in Jamaican men on hormonal therapy for prostate cancer.

## Competing interests

There were no competing interests.

## Authors’ contributions

BM- Study Design, Data collection, statistical analysis, manuscript writing; IB- Data collection and measurements; WA- Study Conception, intellectual content of manuscript; RM- patient recruitment; HF- patient recruitment; intellectual content of manuscript; MR- study design; statistical analysis; manuscript writing.
